# Supplementary material for: Are carbon emissions trading and green financial instruments synergistic? -Comprehensive quantitative research based on content analysis
Source: PLoS One. 2024 Mar 7;19(3):e0298601. doi: 10.1371/journal.pone.0298601 (PMC10919601; doi:10.1371/journal.pone.0298601)
Supplement: S1 Data — (ZIP) [file pone.0298601.s001.zip › Data/地级市Z_F工作报告.zip!/╜¡╦╒/╒≥╜¡╩╨/└┤╫╘╣1⁄2╓┌║┼í╛┬φ┐╦╩2╛▌═°í┐_2008.docx]

各位代表：

　　在党的十七大精神指引下，镇江全面建设更高水平小康社会的新征程即将开启。这次人代会肩负着承前启后、继往开来的重大使命。现在，我代表镇江市人民政府，向大会作工作报告，请予审议，并请市政协各位委员和其他列席人员提出意见。

　　过去五年工作回顾

　　本届政府自2003年组建以来，在中共镇江市委的领导下，认真落实科学发展观，全面建设小康社会，圆满完成各项任期目标，镇江经济社会发展取得显著成就。

　　综合实力实现重大突破。预计2007年，全市地区生产总值完成1213亿元，人均5500美元，分别比2002年增长1.36倍和1.34倍；财政总收入从2002年的56.8亿元增加到2007年的203.3亿元，地方财政一般预算收入从23亿元增加到80.3亿元；全社会固定资产投资完成601亿元，增长2.24倍；社会消费品零售总额完成330亿元，增长1.21倍。累计实际利用外资37亿美元，超过此前历年总和；外贸进出口总额62.5亿美元，增长1.98倍。私个注册资本五年净增335亿元。

　　经济结构取得显著改善。农业结构调整步伐加快，丘陵山区农业综合开发取得成效。五年新增高效农业面积38万亩，实施“三资”开发农业项目1753个，总投资143亿元。坚持工业强市方略，工业规模进一步壮大。全市工业性投入年均增长32.1%，新增销售超10亿元企业21家，50强企业销售占规模以上工业企业销售46.6%。高新技术产品增加值占全市规模以上工业增加值35.1％，比五年前提高11个百分点。国有企业改制基本完成，一批企业通过引进战略投资者做强做大，全市已有9家上市公司。现代服务业发展加快，从2005年开始，服务业增加值增幅连续三年超过地区生产总值增幅。新型商贸业态不断涌现，旅游总收入年均增幅超过20%。以大港三期为重点的港口建设顺利推进，新建、在建万吨级以上泊位15个，港口吞吐量达到7800万吨。

　　城乡面貌发生喜人变化。完成镇江城市总体规划修编，制订了镇江城市分区规划、控制性详规和镇村规划，实现城乡规划全覆盖。城市建设“三年大变样”目标顺利实现，“四年新提升”目标任务全面实施。城市化率达到60.1％，比2002年提高9.2个百分点。市区累计投入城建资金407亿元，“南山北水”建设全面启动，完成南徐新城区和北部滨水区总体规划，建成新城大道、滨江旅游风光带，开工建设拆迁定建房40万平方米，九华山路、南徐路西延、团山路等一批道路工程积极推进，引航道水利枢纽、焦南闸扩建、金山湖整治工程全面展开。完成沪宁高速、312等国、省道扩（改）工程，新建镇溧、宁常高速。城市内外快速交通路网基本形成，五年累计建设对外交通道路459公里、城市道路141公里，建成润扬大桥等一批重大基础设施。实施城市管理重心下移，城管水平不断提高。建成一批农田水利基础设施，累计疏浚县乡河道431条、改造塘坝4500座、治理水土流失面积60平方公里。全面推进新农村建设，农村生产生活条件进一步改善。先后实施两轮农村“五件实事”，新建农村公路2500公里，建制村全部开通客运班车，完成草危房改造和改水、改厕任务，农村卫生院、综合文化站等基础设施建设得到加强。修编完善全市开发区规划，完成基础设施投入135亿元，国家级出口加工区和7个省级开发区有了新的发展。

　　环境保护迈出坚实步伐。自觉践行环保优先，顺应群众呼声，先后开展市区高污染燃料禁燃区专项整治、东部地区大气环境综合整治、312国道两侧环境整治、太湖流域水环境整治、化工行业专项整治和农村环境综合整治等行动。改造搬迁企业40家，关闭高污染、高能耗企业220多家，其中小化工企业120多家，金河纸业实施关浆转产重组，谏壁电厂等一批高耗能、高污染企业得到有效治理。下大力气整治非法开山采石，关闭开采企业156家、采石宕口349个。积极实施“绿色倍增”计划，新增造林面积40万亩，森林覆盖率提高到20.1%。完成国家“863”水环境生态修复示范工程。饮用水水源地保护进一步加强。严格落实节能减排各项措施，单位地区生产总值能耗比2005年下降7.83%，二氧化硫、化学需氧量排放完成省政府下达的削减任务。2007年，空气质量良好以上天数达333天，环境质量综合指数达到80.2。

　　群众生活水平普遍提高。2007年，城镇居民人均可支配收入16750元，比2002年翻了一番；农民人均纯收入从4452元增加到8007元。累计新增城镇就业17.4万人，下岗失业人员再就业10.5万人，转移农村劳动力24.9万人。城镇登记失业率稳定在较低水平，2007年为2.62%。全市城镇养老、医疗、失业保险覆盖面均超过95%，新型农村合作医疗人口参合率达98.8%，在全省率先全部建成“江苏省初级卫生保健先进县（市）”。城乡居民低保标准进一步提高。城镇家庭廉租住房保障制度全部建立，基本实现应保尽保。成立市慈善总会，全市共募集资金2.87亿元，救助困难群众2.3万人次。各项社会事业取得长足进步，市财政用于教育、卫生、文化、社会保障、优抚等公共支出年均增长20％，高于地方一般预算支出1个百分点。坚持教育优先方针，大力推进教育现代化，丹阳、扬中已通过省级教育现代化验收。出台突发公共事件应急总体预案和专项预案，任期内未发生重特大安全生产和食品药品安全事故。坚持每年实施一批为民办实事项目，共改造街巷道路263条，改造老住宅小区160万平方米，治理危房5.9万平方米。

　　政府自身建设不断加强。围绕“创新、服务、高效、规范、清廉”的目标，着力建设人民满意政府。进一步深化行政审批制度改革，累计取消各类审批事项137项、改变管理方式85项。成立市行政服务中心，在全国率先推行以归并行政审批职能为核心的“两集中、两到位”。全市90%的镇（街道）建成便民服务中心，三级便民服务网络初步形成。推进政务公开，建立新闻发言人制度，加强“中国镇江”门户网站建设。在全省率先制订和落实《镇江市政府部门行政首长问责暂行办法》，改进政府服务，规范权力运行，强化责任追究。累计办理人大代表建议1090件、政协提案2137件，办结率达100%，满意率超过96%。五年来，在市委领导下，在市人大、市政协的监督支持下，政府各部门通力协作，全市人民团结一心，形成了政通人和、共谋发展的生动局面。

　　尤其令人振奋的是，在刚刚过去的2007年，全市发展呈现出增长较快、结构优化、效益提高、民生改善的良好态势，主要经济指标达到近几年的最好水平。预计地区生产总值增长15.5%，地方财政一般预算收入增长33.5%；社会消费品零售总额增长18%，城镇居民人均可支配收入、农民人均纯收入分别增长17.2%和19.2%，实际到位外资突破10亿美元。全市总体达到省定小康标准，这是镇江发展进程中的重要里程碑！

　　各位代表，过去五年，是我市改革开放不断深化、综合实力大幅提升的五年，是全面小康建设取得重大进展、人民生活得到更多实惠的五年，是我市对外形象显著提高、发展活力明显增强的五年。五年来，镇江先后荣获国家卫生城市、国家环保模范城市、国家园林城市等称号，继续保持全国科技进步先进市、全国双拥模范城称号。全市自主创新能力综合评价得分在全国地级市中列第9位。社会治安综合治理和“平安镇江”建设连续四年考核总分居全省第一，被命名为全国社会治安综合治理优秀地市。回顾五年，我们深切感受到，每一项成就的取得，都离不开全市人民的参与和支持；每一点发展变化，都凝聚着全市人民的智慧和力量。在此，我代表市人民政府，向为镇江全面小康建设和繁荣进步付出辛勤劳动的全市人民、驻镇部队和武警官兵、广大政法公安干警、驻镇单位干部职工，以及在镇江学习工作、投资创业的海内外朋友，表示衷心的感谢并致以崇高的敬意！

　　总结五年实践，我们深刻体会到，有四条经验必须始终坚持并不断赋予新的内涵：一是始终坚持解放思想、开拓创新。面对国家宏观调控逐渐成为常态，地区间的竞争逐渐转为好、优、快，我们必须着力转变观念、与时俱进，突破固有的思维和路径，用创新的思路和举措迎接新挑战、认识新规律、抢抓新机遇、引领新发展。二是始终坚持科学发展、统筹协调。加大城乡统筹力度，新增财力更多地向“三农”倾斜，努力在发展中逐步缩小城乡差距。在抓好经济建设的同时，必须更加重视生态建设、文化建设、社会建设，走好和谐发展之路。三是始终坚持发展为了人民、发展依靠人民。人民是推动发展的真正动力。我们思考问题要和人民站到一起，重大决策要和人民想到一起，政府??享。四是始终坚持求真务实、争先创优。面对繁重的改革发展任务，全市形成了自加压力、提升标杆，破解难题、竞相发展的生动局面。扬中市于2006年率先建成小康；句容市创成国家卫生城市和国家环保模范城市；丹阳市上市企业达到5家，创成国家级品牌32个，列全省县市第4位；丹徒区创成国家生态示范区；镇江新区去年实际利用外资再攀新高，达到3.1亿美元；京口区、润州区立足主城区，现代服务业取得长足发展，财政总收入均突破10亿元。

　　在肯定成绩的同时，我们也清醒地看到发展中的问题与不足：综合经济实力和区域竞争力还不够强，经济结构性矛盾依然比较突出，产业结构偏重、产业层次偏低、消耗排放偏高，企业核心竞争力和科技创新能力不强，新型工业化步伐还不快；农业持续增效、农民持续增收、农村持续发展的长效机制还没有形成，文化建设、社会建设相对滞后于经济建设；老城区危旧房改造、困难群体就业、提高社会保障水平等方面，还有大量工作要做；转变政府职能、改善公共服务，还需要进一步加强。对这些问题，必须高度重视，切实加以解决。

　　今后五年目标任务

　　各位代表，历经30年改革开放，镇江正站在新的发展起点上。今后五年，是镇江全面建设更高水平小康社会的重要时期，是优化发展、加速提升的关键阶段。镇江能否在过去成绩的基础上续写新的辉煌，关键看这五年；能否在激烈的区域竞争中创造优势、彰显特色，关键看这五年；城乡面貌能否有巨大变化，人民群众幸福感能否明显提升，关键看这五年！全市上下务必抓住机遇，开拓创新，以奋进的精神状态和饱满的工作热情，全力推进镇江朝着更加富裕繁荣、更加清新秀美、更加开放文明、更加和谐安宁、更加充满活力的目标阔步前进，率先建成更高水平的小康社会。

　　——奋斗五年，镇江经济更加繁荣，产业特色更加鲜明，综合竞争力显著增强。到2012年，全市地区生产总值超过2400亿元，人均1万美元以上，双双实现翻一番。着力优化结构、提升层次，实现产业结构由传统产业为??集约型的双重转变。加快发展高新技术主导的先进制造业。按照主导产业高端化、新兴产业规模化、传统产业高新化的思路，举全市之力，做大做强太阳能光伏、半导体照明和功能纤维三大新兴产业，分别突破百亿规模；现代装备制造业突破千亿规模，新医药、电力电气产业双双超过500亿规模，高新技术产品增加值占到规模工业增加值的44%以上。加快发展高端产业引领的现代服务业。引进和培育一批服务外包、现代物流等高端服务企业，力争服务外包营业总收入达到250亿元，早日建成亿吨大港，现代物流业增加值占到地区生产总值的8%。服务业增加值占地区生产总值的比重达到42%。加快形成高新产业集聚的新园区。集中力量推进大学科技园、服务外包基地和软件园三大园区建设，将丁卯经十二路沿线建成高新产业特色鲜明、拥有自主知识产权、领军型创新人才集聚的高新技术产业带。

　　——奋斗五年，镇江城乡面貌焕然一新，展现出集自然山水之美与现代都市之美于一体、古代文明与现代文明交相辉映的独特魅力。统筹城乡建设，着力推进城乡一体化。按照省委提出的“五个一体化”要求，加快形成以城带乡、城乡共同发展的长效机制。“一体两翼”城市格局加快形成，城镇化率提高到66%。投资250亿元，全力打造“南山北水”：让钟灵毓秀的南山尽展新城风采，怀抱长江的北部滨水区尽显古城神韵。加快老城区危旧房成片改造，着力解决“城中村”问题，建设优美人居环境。全面推进社会主义新农村建设，基本达到“生产发展、生活宽裕、乡风文明、村容整洁、管理民主”的要求。对外交通更加快捷畅达，基本形成通港（空港、江港）、通园（开发园区）、通区（工业集中区）的快速交通系统，所有镇15分钟内驶上快速干道。抓住京沪高速铁路、沪宁城际轨道和泰州长江大桥建设的机遇，进一步凸显镇江的综合交通枢纽优势。发展文教事业，彰显名城人文新特色。加快文化体制改革步伐，从提升软实力的高度，积极构建较为完备的公共文化服务体系和文化产业发展体系。加强名城保护和开发，弘扬镇江优秀传统文化，传承创新民间特色工艺，大力发展文化创意、文化博览、广播影视、文艺演出、动漫游戏等文化产业，推动文化大发展、大繁荣。文化及相关产业增加值增幅明显高于同期经济增幅，力争到2012年占地区生产总值的比重达到5%以上，文教娱乐及服务支出占家庭消费支出比重保持在18%以上。坚持教育优先方针，加大教育投入，努力建设人民满意教育，全面实现教育现代化。加大环保力度，营造宜居生活新环境。到2012年，森林覆盖率提高到23%以上，建成区绿化覆盖率达到45%，空气优良天数占到全年90%以上，环境质量综合指数超过85。铺设老城区雨污分流管网100公里，城市生活污水处理率达到90%以上，集镇建成区污水处理率达70%以上，农村生活污水处理率达40%以上。生活垃圾全部实现集中处理。区域供水率达到100%，实现镇村自来水全覆盖。创建国家生态市，建成国家生态园林城市。

　　——奋斗五年，镇江人民生活更加幸福，社会更加和谐。城乡就业更加充分。统筹城乡劳动就业，改善农民进城就业待遇，推进统一的城乡人力资源市场建设，加大政府购买公益性岗位力度，为更多的困难群体提供就业援助。五年新增城镇就业15万人、转移农村劳动力10万人、培训农村劳动力10万人，城镇登记失业率控制在3%左右。收入增长机制更加健全。建立与经济增长相适应的职工工资增长机制，促进职工收入随企业效益同步增长。强化工资支付保障机制，提高低收入者的工资性收入，促进社会分配公平。刚性落实最低工资保障制度，提高最低工资标准。创造条件让更多群众拥有财产性收入。城镇居民人均可支配收入、农民人均纯收入年均分别增长12%和11%以上。各类保障更加完善。城乡居民各项社会保障覆盖面达到95%以上，农村新型合作医疗覆盖面稳定在98%以上。积极顺应人口老龄化趋势，大力发展养老服务业，初步建立以居家养老为基础、社区服务为依托、社会养老为补充的养老机制。建立新型农村养老保险制度，实现城乡养老保险全覆盖，对城乡无收入老年居民，按照低标准起步、逐年增加的要求，政府予以补贴。提高城乡低保标准，确保每人每天生活费超过1美元，彻底消除绝对贫困现象，并随着人民群众生活水平的提高而增长。完善住房保障制度，努力使低保家庭住得上廉租房，低收入家庭住得起经济适用房，新就业人员租得起房。

　　各位代表，未来五年，目标宏伟，任务艰巨。发展有新要求，人民群众有新期待，新一届政府承载着光荣使命，一定要保持清醒头脑，自觉服从和积极顺应国家宏观调控。既要充分看到发展的有利条件和面临的现实机遇，增强机遇意识，坚定工作信心；又要充分估计发展中可能出现的困难和挑战，增强忧患意识，始终居安思危，更加积极地抓住机遇，更加有效地破解难题。我们坚信，有市委的正确领导，有各位代表和委员的共同努力，有300万镇江人民团结拼搏，勤劳和智慧一定能结出累累硕果，今天绘就的蓝图一定会在奋斗中成为现实！

　　2008年政府工作建议

　　2008年是全面贯彻落实十七大精神的第一年，也是新一届政府施政的开局之年。做好今年工作，对于实现今后五年的目标，赢得发展先机，具有十分重要的意义。按照市委五届五次全会的要求，政府工作的指导思想是：以党的十七大精神为指导，深入贯彻落实科学发展观，准确把握宏观形势，坚持优化发展，推进加速提升，加快转变经济发展方式，着力优化产业结构，提高开放层次，保护生态环境，切实改善民生，促进社会和谐，更好更快推进“全面达小康，建设新镇江”。

　　全市经济社会发展主要预期目标是：地区生产总值增长13.6%；单位生产总值能耗下降4%，二氧化硫、化学需氧量排放分别削减4%和2%；财政总收入增长16%，地方财政一般预算收入增长16%；全社会固定资产投资增长18%；社会消费品零售总额增长15%；城镇居民人均可支配收入增长12%，农民人均纯收入增长10%；城镇登记失业率控制在3.5%以内。其中，节能减排指标是约束性的，必须作为硬任务，千方百计确保完成；其他指标是指导性的，力争在工作中完成得更好。

　　围绕上述目标要求，重点做好以下七个方面的工作：

　　（一）加快富民惠民步伐，切实改善民生

　　大力促进全民创业。进一步落实鼓励创业的各项政策，培育创业文化，营造创业环境，不断浓厚全民创业氛围，使更多的劳动者成为创业者。提高小额贷款效率，搭建创业服务平台，建设中小企业创业基地。积极开展多层次、多种形式的技能培训，继续推进“百人千企”创业辅导活动。

　　统筹城乡充分就业。认真落实就业优惠政策，实施就业“绿色通道”制度。通过政府购买公益性岗位等途径，重点对零就业家庭、“4050”、下岗失业人员、被征地农民、残疾人等开展就业援助。建立城乡统一的就业、失业登记制度，对农村富余劳动力实行动态管理。继续开展充分就业创建活动，力争3个以上辖市（区）实现充分就业，城镇充分就业社区创建率达98%以上，农村劳动力充分转移镇、村创建率达95%以上。贯彻落实《劳动合同法》，提高劳动合同覆盖率，全面推进劳动监察网格化管理，切实维护职工合法权益。

　　完善社会保障体系。着力构建城乡一体化的社会保障体系。进一步加大扩面征缴工作力度，确保全市基本养老保险新增5万人，各类社会医疗保险参保242万人，提前实现覆盖90%以上城乡人口的“人人享有社会医疗保障”目标。巩固提高职工基本养老、医疗、失业保险制度，全面建立新型农村养老保险制度、被征地农民即征即保制度，完善城镇居民医疗保险制度。继续实行社区卫生服务机构药品“零差率”政策，减轻参保群众医药费用负担。健全城乡低保标准增长机制，完善物价上涨动态补贴制度。进一步发展慈善事业，不断扩大各级慈善机构的基金规模和救助能力，完善社会救助制度。

　　今年，为民办好15件实事：

　　1．新增城镇就业3万人，转移农村劳动力3万人，培训农村劳动力3万人。

　　2．建立城乡老年居民养老补贴制度；完善医疗救助制度，对特殊困难人员提供免费的基本医疗社区门诊服务；完成市区10万名企业退休人员的健康体检。

　　3．财政新增农村新型合作医疗、居民医疗补贴3500万元（总量超亿元），城镇居民医保参保率达90%以上，农村新型合作医疗参合率稳定在98%以上。

　　4．新建林隐路、丁卯2个菜市场，升级改造五条街、牌湾、京口闸等8个菜市场；在城郊新建蔬菜基地2000亩。

　　5．改造塔山桥、燕舞桥，改造农村公路危桥20座。

　　6．治理烈士陵园周边、东吴路74号南侧山体滑坡；整治改造老住宅小区30万平方米；整治周家河新村、光明村、梳儿巷16号等低洼积水区；改造市区街巷道路2万平方米；改造危房2万平方米；实施中华路、京畿路等地段路灯改造。

　　7．开工建设经济适用房20万平方米，其中竣工10万平方米；新建廉租住房5500平方米。

　　8．建设南徐新城公交停车场；新增环保节能公交车80辆，新辟和优化公交线路8条，城市公交有计划地向丹徒区、镇江新区各镇延伸。

　　9．建成全民健身工程点20个，农村建成小篮球架1000个；维修更新市区健身器材。

　　10．为1.5万对新婚夫妇提供健康教育服务，降低新生儿出生缺陷发生率，确保孕前准备及指导普及率达85%以上。

　　11．建设残疾人综合服务中心；建成残疾人庇护所；建设县级残疾人康复中心3个。

　　12．50%的建制村建成社区服务中心；建设为农服务社550个。

　　13．市区新建成停车场3个，增加公共停车泊位1500个。

　　14．区域供水覆盖到全市各镇。

　　15．建立和完善法律援助工作保障机制，扩大援助覆盖面，援助案件突破1000件。

　　（二）推进产业结构调整，促进优化升级

　　坚持一手抓先进制造业集聚，一手抓现代服务业发展，加快产业结构调整和优化升级，增强经济竞争力。

　　加快新型工业化步伐。优化投资结构，完成工业投入470亿元，全力抓好143个亿元以上、投资总额达846亿元的重点项目。培育规模企业，力争超亿元企业达到340家，超10亿元企业达到28家。发展壮大三大新兴产业，打造新的增长点。着力培育光伏产业，加大投入尽快形成产业链；增强半导体照明产业的应用能力，扩大产业规模；推进功能纤维项目的建设与产业化。根据船舶产业发展规划，加快在建、在手、在谈造船项目推进，使之尽快成为优势产业。积极运用新技术、新工艺和信息化，改造提升传统产业，提高产品附加值和产业竞争力。大力推进品牌战略，争创省级以上品牌40个以上。

　　加快发展现代服务业。提升传统商贸业态，大力发展连锁超市、购物中心、特色商业街等新型业态。加快推进市场建设，确保第一楼街步行街、长江新天地、镇江国际工业品城等重点市场建成开业。开工和续建码头泊位13个，大力发展港口物流和集装箱运输，促进生产性服务业的发展。做大做强旅游产业，以重大旅游项目建设为抓手，推进旅游联动开发，建设乡村旅游精品，创建1－2家全国农业旅游示范点。加快推进世业洲东湖景区等项目建设。把发展服务外包、软件产业作为新兴服务业的重中之重，大力培育和引进市场主体，加快服务外包基地和软件园建设，力争引进3－5家国际服务外包企业。做大做强金融保险业，积极引进新兴的金融机构，打造良好的金融生态环境。发展“普惠金融”，重点加大对县域、“三农”、高新技术产业和中小企业信贷支持力度。规范发展房地产业。创新社区服务模式，政府主导、多元投入，积极拓展社区养老、卫生、教育等服务项目，提升服务水平。

　　大力提升自主创新能力。坚持走以应用研发为特点的自主创新之路，强化企业在自主创新中的主体地位、在产学研结合中的主导作用。新增省级高新技术企业50家，高新技术产品100项。新增国家、省、市技术中心15家以上。高新技术产品增加值占全市规模以上工业的37%以上。专利申请量超过5000件。加大科技成果转化力度，组织实施省级以上科技计划项目100项。进一步整合资源，加快镇江留学人员创业园、省级大学科技园、科技创业服务中心等平台建设。大力实施人才强市战略，加快人才引进和培养步伐，重点引进拥有创新成果、通晓国际先进管理和商务规则、善于运作科技资源的创新创业领军人才，力争有更多的人才进入省“高层次创业创新人才引进计划”。引导支持更多企业建立博士后工作站。

　　（三）加快发展现代农业，繁荣农村经济

　　解决好“三农”问题，促进新农村建设，事关全面建设小康社会大局。全市各级都要继续关注农业、关心农民，重视农村发展。加大财政支农惠农力度，全市财政安排支出5.08亿元，同比增长26.7%。

　　加快高效农业规模化。把高效农业规模化作为推动现代农业发展的着力点，进一步优化“三大板块”、“七条走廊”的区域布局，促进134个高效农业重点项目基地化。新增亩均效益2000元以上的高效农业面积15万亩，其中亩均效益5000元以上的5万亩。全力实施品牌战略，提升农产品质量安全水平，大宗农产品抓“无公害”，特色农产品抓“绿色”，优势农产品抓“有机”，新增“三品”认证30个。提升现代农业机械装备水平，增强农业综合生产能力。落实最严格的土地管理和耕地保护制度，加大土地开发整理复垦力度，加强基本农田标准化建设。

　　创新农业发展机制。坚持“依法、自愿、有偿”原则，积极推进并规范土地承包经营权流转，发展多种形式的适度规模经营。按照“四有”合作经济组织的要求，引导各类投资主体兴办、领办或合办农民专业合作经济组织，开展农民专业合作社试点，推广“龙头企业＋合作经济组织＋农户”的一体化经营，新增各类农民合作经济组织40家。完善“百村千户”挂钩帮扶机制，积极化解村级债务。

　　继续办好农村新五件实事。新建农村公路200公里。继续扶持经济薄弱地区乡镇卫生院服务能力建设、乡镇综合文化站建设和有线电视进村入户工程。推进新一轮农村改水、河塘疏浚工程，加强中小型水库除险加固工程建设。

　　（四）深化改革开放，增强发展活力

　　加大改革力度。完善法人治理结构，促进改制企业更好发展。推动企业股份制改造，培育更多的上市后备企业。鼓励有条件的企业在境内外上市，年内新增上市企业3家以上，支持发行企业债券和已上市企业再融资。继续深化投资体制改革，积极引进风险投资、创业投资基金，运用私募、信托等手段拓宽资金渠道。强化国有资本吸纳，引进更多的国有大企业来镇投资。进一步深化市区城管体制和财政体制改革，增强市区政府提供公共服务的能力。大力发展民营经济。健全资本、土地、技术等要素市场，培育发展担保机构，为民营企业创造良好发展环境。全年吸纳民资实际到位数160亿元，新增私营企业3300家、个体工商户1.3万家、注册资本55亿元。

　　提升开放型经济水平。优化利用外资结构，变招商引资为招商选资、招商引智。瞄准世界500强和跨国公司，引进一批高附加值、高技术、高税收的大项目，促进开放型经济质量整体提升，全年实际到位外资增长15%以上。积极实施产业链招商，重点引导外资更多地投向高端制造和研发环节，投向节能环保产业、现代服务业和现代农业。在更高水平“引进来”的同时，更大步伐地“走出去”。支持出口企业开发拥有自主知识产权和核心技术的产品，进一步提高机电产品、高新技术产品的出口份额。培育发展外经重点产业和重点企业，积极扩大工程承包和劳务合作。

　　提升园区承载能力。着力在培育园区产业特色、提升产业配套能力、完善基础设施功能等方面取得新突破，促进开发园区成为先进产业的集聚区、科技创新的先导区、节能环保的示范区。进一步完善国家级镇江出口加工区功能，推进丹阳、句容出口加工区申报工作。坚持节约集约规划和建设好乡镇工业集中区。省级开发区基础设施投入确保20亿元以上，n>

　　（五）完善城市功能，统筹城乡建设

　　加快推进“南山北水”建设。南徐新城区全面完成征地工作，开工建设行政中心区、规划展示馆，建设黄山中路等5条道路。加快凤凰家园、新城市花园、九华山庄安置房小区及配套设施建设，争取年内竣工30万平方米。北部滨水区基本完成引航道水利枢纽、焦南坝修复和金山湖景区建设工程。

　　完善城市功能。按照规划引导、先易后难的思路，开展“城中村”和老城区危旧房成片改造试点。开工建设大市口中心区地下过街通道。推进城东垃圾填埋场二期续建工程。改造老城区合流管道20公里，改造和建设征润洲、丁卯、谏壁、大港和丹徒等污水处理和收集系统，城市生活污水处理率达到84.5%。编制历史文化名城控制性详规，实施西津渡历史文化街区保护更新二期工程。全面提升城市管理水平，推进城市管理数字化、环卫作业机械化、垃圾收集分类化，加强市区卫生保洁全覆盖管理。创建1－2条市容管理示范路，继续有序推进市区夜景亮化、美化建设。

　　统筹城乡基础设施建设。以实施新的《城乡规划法》为契机，完善市域城乡规划。进一步优化城乡路网框架，宁杭高速镇江段和243省道句容段建成通车，续建和改建241、338、122和238省道。开工建设京沪高速铁路镇江段和泰州长江大桥（经扬中）。各辖市要进一步加大城市建设力度，提升城市形象，完善城市功能，增强对镇村的辐射。加强重点中心镇建设，加快城镇化步伐。

　　（六）坚持环保优先，努力建设生态文明

　　切实加强节能减排。实行更加严格的环境准入制度，严格限制新上污染项目，坚决淘汰落后工艺，改造和关停一批严重污染企业。进一步强化节能减排工作责任制，认真开展污染源普查工作，突出抓好重点行业、重点企业、重点工程的节能减排工作。抓好5家省级循环经济试点，突出做好全市5000吨以上标煤企业的节能降耗工作。推广清洁生产和可再生资源利用，切实提高资源利用率，降低能耗排放量，确保完成年度总量削减目标。

　　深入开展专项治理。巩固和提高小康社会环境质量综合指数。加强太湖流域水污染治理工作，相关各镇全部按期建成污水处理厂。全面实施污染物排放新标准，严格治理河道排污。加强饮用水水源地保护。深入推进化工行业专项整治，加快化工集中区建设。全面完成矿山关闭任务，引入市场化机制，推进宕口复垦复绿综合整治工作。深入开展城乡环境综合整治，突出抓好城市生活污染和农村面源污染治理。

　　进一步推进生态建设。继续大力开展绿化造林活动，提高城乡绿化的生态效果和景观效果。全市新增造林面积13万亩，市区新增绿地100公顷。丹阳市、扬中市通过国家环保模范城市考核验收，丹阳市建成国家生态示范区。做好市区创模复查迎检工作。启动国家生态园林城市创建工作。

　　（七）加快发展社会事业，构建和谐社会

　　深入推进文化建设。加强社会主义核心价值体系建设，发挥先进思想文化的引领作用，开展各类精神文明创建活动，不断提高全民文明素质。大力繁荣文化事业，进一步整合文化资源，完善文化设施，培育文化市场，保护利用历史文化遗产，创作一批与本土文化紧密结合的作品。推进新广电中心、体育会展中心等公共文化基础设施建设，完成文化艺术中心设计，全面实现“辖市（区）有两馆、镇（街道）有一站、村（社区）有一室”目标，丰富城乡居民的文化生活。积极发展文化产业，力争在文化创意产业发展上实现突破。

　　加快发展社会事业。进一步调整和优化财政支出结构，新增财力主要向关系民生的社会发展薄弱环节倾斜，今年市财政用于教育、卫生和环保等公共事业的支出增长27.8%。优先发展教育事业，更加注重教育的普惠性，合理配置公共教育资源，促进义务教育均衡发展，所有辖市（区）基本实现教育现代化。城乡义务教育全部免费提供课本。积极发展与镇江产业需求相适应的职业技术教育。积极支持在镇高校建设。推进医疗卫生体制改革，合理配置卫生资源，加快城乡卫生服务体系建设，努力为群众提供安全、有效、方便、价廉的医疗卫生服务。建成市急救中心并投入使用，提高对重大疾病防控和突发公共卫生事件应急处置能力。积极开展全民健身活动，努力??、外事、侨台、气象、防震减灾、妇女儿童等工作。

　　加强民主法制和“平安镇江”建设。自觉接受人大、政协监督，高度重视办好人大议案、代表建议和政协提案。进一步发扬社会民主，完善村民自治、社区居民自治和企业职工代表大会等民主管理制度，保障基层群众依法行使民主权利，维护社会公平正义。把社会管理放在更加突出的位置，推进管理重心下移，强化街道社区的社会管理和服务居民职能。深入开展“平安镇江”、“法治镇江”创建工作。全面落实社会治安综合治理各项措施，完善城乡社会治安防控体系，加强流动人口服务和管理，依法严厉打击各种刑事犯罪。进一步推进“五五”普法，增强全社会的法制意识。完善高效统一的应急指挥体系，不断增强预防和处置突发公共事件的能力。全面落实安全生产责任制，坚决防止重特大事故发生，切实加强食品、药品安全及公共场所卫生监督管理工作。加强国防后备力量建设，积极开展双拥“六创”工作。完善“大调解”机制，进一步做好人民群众来信来访工作，重视解决人民群众合理诉求，有效化解人民内部矛盾，努力使社会更加和谐安宁。

　　加强政府自身建设

　　面对新的形势、新的任务，新一届政府必须以崭新的面貌、崭新的理念、崭新的作为，切实加强自身建设，不断提高管理经济社会事务的能力和水平。

　　进一步振奋精神。在任何情况下，良好的精神状态，都是事业心、责任心的直接体现，是战胜困难、取得突破的重要保证。全体公务人员一定要以对镇江发展高度负责、对人民群众高度负责的态度，敢于直面挑战，敢于迎难而上，恪尽职守，勇挑重担，创造出无愧于历史、无愧于时代、无愧于人民的新业绩。

　　进一步解放思想。要按照科学发展的新理念来解放思想，根据发展阶段的新变化来调整思路，顺应人民群众的新期待来转变观念，自觉摒弃不合时宜的惯性思维和束缚发展的陈旧观念。大力倡导自觉学习、终身学习，善于在实践中提高领导科学发展的能力、研究新情况和解决新问题的能力、统筹兼顾的能力、抓好落实的能力，以宽广的视野、创新的思路，不断开创各项工作的新局面。

　　进一步提升效能。紧紧围绕建设现代服务型政府的目标，进一步深化行政管理体制改革，加快转变职能。规范行政审批，加强市行政服务中心建设，强化社会管理和公共服务，完善公共服务体系。深入推进政务公开，加快电子政务建设，实现政府新闻发布制度常规化，确保权力在阳光下运行。坚持依法行政、从严治政、廉洁从政。严格落实党风廉政建设责任制，完善惩治和预防腐败体系。强化效能监察和行政问责，严肃查处行政不作为和乱作为，坚决纠正损害群众利益的各种不正之风。加强绩效评估，切实提高工作效率和服务水平。

　　进一步改进作风。大力弘扬艰苦奋斗的优良作风，带头厉行节约，勤俭办事，力戒形式主义，反对排场和浪费，努力降低行政成本。腾出更多的时间到基层去，到群众中去，到困难多、问题多的地方去，深入调查研究，多做打基础、管长远的工作，多办得民心、顺民意的事情，踏踏实实地干，开拓创新地干。努力使政府的各项工作与群众的需求一致起来，各项目标与群众的意愿一致起来，营造政通人和、心齐气顺、团结奋进的良好氛围。

　　各位代表，实现未来五年的发展目标，需要全市人民的共同努力。让我们高举中国特色社会主义伟大旗帜，以党的十七大精神为指引，在中共镇江市委的正确领导下，深入贯彻落实科学发展观，团结一致，与时俱进，开拓创新，奋发图强，为全面建设更高水平的小康社会而努力奋斗！
